# Fatty acid desaturase (*FADS*) gene polymorphisms and insulin resistance in association with serum phospholipid polyunsaturated fatty acid composition in healthy Korean men: cross-sectional study

**DOI:** 10.1186/1743-7075-8-24

**Published:** 2011-04-23

**Authors:** Oh Yoen Kim, Hyo Hee Lim, Long In Yang, Jey Sook Chae, Jong Ho Lee

**Affiliations:** 1 Yonsei University Research Institute of Science for Aging, Yonsei University, Seoul, Korea; 2 Clinical Nutrigenetics/Nutrigenomics Lab, Dept of Food and Nutrition, Yonsei University, Seoul, Korea

**Keywords:** *FADS *gene, homeostasis model assessment-insulin resistance, linoleic acid, dihomo-γ-linolenic acid, arachidonic acid

## Abstract

**Background:**

We investigated the relationship between fatty acid desaturase (*FADS*) gene polymorphisms and insulin resistance (IR) in association with serum phospholipid polyunsaturated fatty acid (FA) composition in healthy Korean men.

**Methods:**

Healthy men (n = 576, 30 ~ 79 years old) were genotyped for rs174537 near *FADS1 *(*FEN1*-10154G>T), *FADS2 *(rs174575C>G, rs2727270C>T), and *FADS3 *(rs1000778C>T) SNPs. Dietary intake, serum phospholipid FA composition and HOMA-IR were measured.

**Results:**

Fasting insulin and HOMA-IR were significantly higher in the rs174575G allele carriers than the CC homozygotes, but lower in the rs2727270T allele carriers than the CC homozygotes. The proportion of linoleic acid (18:2ω-6, LA) was higher in the minor allele carriers of *FEN1*-10154G>T, rs174575C>G and rs2727270C>T than the major homozygotes, respectively. On the other hand, the proportions of dihomo-γ-linolenic acid (20:3ω-6, DGLA) and arachidonic acid (20:4ω-6, AA) in serum phospholipids were significantly lower in the minor allele carriers of *FEN1*-10154 G>T carriers and rs2727270C>T than the major homozygotes respectively. AA was also significantly lower in the rs1000778T allele carriers than the CC homozygotes. HOMA-IR positively correlated with LA and DGLA and negatively with AA/DGLA in total subjects. Interestingly, rs174575G allele carriers showed remarkably higher HOMA-IR than the CC homozygotes when subjects had higher proportions of DLGA (≥1.412% in total serum phospholipid FA composition) (*P *for interaction = 0.009) or of AA (≥4.573%) (*P *for interaction = 0.047).

**Conclusion:**

HOMA-IR is associated with *FADS *gene cluster as well as with FA composition in serum phospholipids. Additionally, HOMA-IR may be modulated by the interaction between rs174575C>G and the proportion of DGLA or AA in serum phospholipids.

## Backgrounds

It has been reported that fatty acid (FA) composition in serum phospholipids as well as a high fat intake might influence the insulin sensitivity and the progression of obesity [1-3]. People with insulin resistance (IR) or metabolic syndrome (MetS) had high levels of palmitic acid (16:0, PA) and low levels of linoleic acid (18:2ω-6, LA) in serum phospholipids [4,5]. Lopes-Alvarenga et al [1] showed that serum triglyceride (TG) level in MetS subjects was positively associated with saturated FAs (SFA), but negatively with long-chain polyunsaturated fatty acids (PUFA). According to Leeson et al [5], higher proportions of docosahexaenoic acid (22:6ω-3, DHA) in erythrocyte lipids were associated with the improved endothelial function, particularly in young smokers who had some features of the IR.

FA composition in serum lipid esters is known to reflect the dietary FA composition during recent one or two months [6-9]. Serum PUFA compositions are influenced by both the FA metabolisms and the genetic variations [10-12]. The key enzymes involved in PUFA metabolism are the Δ5 desaturase (D5D) and Δ6 desaturase (D6D) which are encoded by the FA desaturase 1 (*FADS1*) and *FADS2 *genes located on chromosome 11 (11q12-13.1) [13-16]. This gene cluster also includes a *FADS3*, another desaturase gene which shares 52 and 62% sequence of the *FADS1 *and *FADS2 *genes, respectively [13], but the activity has not been fully identified yet. Among the single nucleotide polymorphisms (SNPs) in *FADS *gene cluster, rs174537 (*FEN1*, flapstructure specific endonuclease) near *FADS1 *shows the most significant association with FAs in a genome-wide association study. Minor T allele homozygotes of the rs174537 (*FEN1*-10154G>T) are associated with lower levels of plasma arachidonic acid (20:4ω-6, AA) and higher levels of LDL cholesterol and total cholesterol than the major G allele homozygotes [16].

Recently, Dupis et al. [17] and Ingelsson et al [18] reported that *FADS*1 (rs174550) was strongly associated with fasting glucose, fasting insulin, homeostasis model assessment of IR (HOMA-IR), HOMA-beta cell function and type 2 diabetes in Caucasian population. However, there were no studies on the relationship between *FADS *gene and IR in association with serum PUFA composition in healthy Koreans. Therefore, this present study aimed to investigate whether *FADS *gene polymorphisms are associated with IR as well as with serum phospholipid FA composition in healthy Korean men.

## Subjects and Methods

### Study population

Study participants (men) were recruited from the Health Service Center (HSC) in the course of a routine checkup visit or by a newspaper announcement for health examination. Subjects were excluded if they have orthopedic limitations, weight loss/gain over the previous 6 months, or any diagnosis of vascular disease, diabetes mellitus, cancer (clinically or by anamnesis), renal disease, liver disease, thyroid disease, and acute or chronic inflammatory diseases. None of all subjects were taking any medications (antihypertensive, antidyslipidemic, antithrombotic and antidiabetic drugs). Finally, 576 healthy men (aged from 30 to 79 years old) were enrolled in the study. Written informed consent was obtained from all subjects, and the protocol was approved by the Institute of Review Board of Yonsei University.

### Anthropometric parameters and blood collection

Body weight and height were measured unclothed and without shoes in the morning. Body mass index (BMI) was calculated as body weight in kilograms divided by height in square meters (kg/m^2^). Blood pressure (BP) was obtained from the left arm of seated patients with an automatic blood pressure monitor (TM-2654, A&D, Tokyo, Japan) after 20 min of rest. Study subjects were interviewed for their smoking and drinking behavior at their visit. Smoking habit was categorized to 'current smoker' and 'non-smoker', and drinking habit was also categorized to 'current drinker (current alcohol consumption)' and 'non-drinker'.

After overnight fast, venous blood specimens were collected in EDTA-treated and plain tubes. The tubes were immediately placed on ice until they arrived at the analytical laboratory (within 1-3 h). Then, the blood specimens were separated into plasma (i.e. glucose and insulin) or serum (i.e. lipid profiles, apolipoproteins and fatty acid compositions), and stored at -70°C until analysis.

Genomic DNA was extracted from 5 mL whole blood using a commercially available DNA isolation kit (WIZARD^R ^Genomic DNA purification kit, Promega, Madison, WI, USA) according to the manufacturer's protocol.

### Genotyping of *FADS *gene cluster

Genomic DNA was extracted from 5 mL whole blood using a commercially available DNA isolation kit (WIZARD^R ^Genomic DNA purification kit, Promega, Madison, WI, USA) according to the manufacturer's protocol. Based on the previous reports of genetic studies as well as the public databases on the *FADS *gene cluster [16,19-21] and HapMap project, http://www.hapmap.org), 8 relevant *FADS *single nucleotide polymorphisms (SNPs) were pre-screened in randomly selected 217 subjects [rs174537 (*FEN1*-10154G>T near *FADS*1), rs174575 (*FADS*2), rs1000778 (*FADS*3), rs2727270 (*FADS*2), rs174576 (*FADS*2), rs174570 (*FADS*2), rs174583 (*FADS*2), rs174456 (*FADS*3)]. Each genotyping reaction was performed with Taqman assay (Applied Biosystems, Foster City, CA, USA).

### Serum Lipid profile, Apolipoprotein A1 and B

Fasting serum total cholesterol and TG were measured using commercially available kits on a Hitachi 7150 Autoanalyzer (Hitachi Ltd. Tokyo, Japan). After precipitation of serum chylomicron, low density lipoprotein (LDL) and very low density lipoprotein (VLDL) with dextran sulfate-magnesium, high density lipoprotein cholesterol (HDL-C) left in the supernatant was measured by an enzymatic method. LDL cholesterol (LDL-C) was estimated indirectly using the Friedewald formula for subjects with serum TG concentrations <400 mg/dL (4.52 mol/L). In subjects with serum TG concentrations ≥400 mg/dL (4.52 mol/L), LDL-C was measured by an enzymetic method on a Hitachi 7150 Autoanalyzer directly. Each sample was measured duplicate and their average value was used for one value. If there is big variation between the two values, the samples were remeasured. Serum apolipoprotein (Apo) A1 and B were determined by turbidometry at 340 nm using a specific anti serum (Roche, Basel, Switzerland).

### Glucose, Insulin and HOMA-IR

Fasting plasma glucose was measured by a glucose oxidase method using the Glucose Analyzer (Beckman Instruments, Irvine, CA, USA). Plasma Insulin was measured by radioimmuno-assays with commercial kits from Immuno Nucleo Corporation (Stillwater, MN, USA). Insulin resistance (IR) was calculated with the homeostasis model assessment (HOMA) using the following equation: IR = {fasting insulin (μIU/ml) × fasting glucose (mmol/l)}/22.5 [22].

### Fatty acid composition in serum phospholipids

Serum phospholipid fatty acid (FA) composition was analyzed using the modified method of Folch et al. [23] and Lepage et al. [24] with gas chromatography (Hewlett Packard 5890A, CA, USA). Individual FAs were calculated as a relative percentage with the elevated FAs set at 100% using Chemstation software.

### The assessment of dietary intake/physical activity level

The subjects' usual diet information was obtained using both a 24-hour recall method and a semi-quantitative food frequency questionnaire (SQFFQ) of which the validity had been previously tested [25]. We used the former to carry out analyses and the latter to check if the data collected by 24-hour recall methods was representative of the usual dietary pattern. All subjects were given written and verbal instructions by a registered dietitian on completion of a 3-day (2 week days and 1 weekend) dietary record. Dietary energy values and nutrient content from a 3-day food records were calculated using the Computer Aided Nutritional analysis program (CAN-pro 2.0, Korean Nutrition Society, Seoul, Korea). Total energy expenditure (TEE) (kcal/day) was calculated from activity patterns including basal metabolic rate, physical activity for 24 hours [26], and specific dynamic action of food. Basal metabolic rate for each subject was calculated with the Harris-Benedict equation [27].

### Statistical analysis

Statistical analyses were performed with SPSS version 15.0 for Windows (Statistical Package for the Social Science, SPSS Ins., Chicago, IL, USA). Hardy-Weinberg Equilibrium (HWE) and the linkage disequilibrium (LD) tests were examined using the Haploviewer 4.1 (Broad Inst, MA, USA). In order to test the difference in general characteristics and biochemical parameters (ie. lipid profiles, glucose, insulin resistance, fatty acid composition and dietary intakes), student t-test (independent t-test) between the two groups (major allele homozygotes vs minor allele carriers) and one-way analysis of variance (ANOVA) among the genotype groups followed by *Bonferroni *correction for multiple comparisons to reduce the rate of false positive was used. General linear model analysis (GLM, Post hoc multiple comparison tests) followed by *Bonferroni *correction for multiple comparisons was also performed to see the differences in IR and FA composition between genotype groups with adjustment for confounders (i.e. age, body mass index, cigarette smoking, alcohol drinking, blood pressure, triglyceride, total energy intake and expenditure etc.). The *Pearson *correlation analyses were used to evaluate the relationship between insulin resistance (HOMA-IR) and general or biochemical parameters including FA composition. In addition, multiple stepwise regression analysis was performed to see whether *FADS *SNPs remain significant influent factor on HOMA-IR together with other markers related to HOMA-IR. Frequencies were tested by chi-square test. We determined whether each variable was normally distributed before statistical testing, and logarithmic transformation was performed on skewed variables. For descriptive purposes, mean values are presented using untransformed values. Results are expressed as means ± standard deviation (S.D.) or %. A two-tailed value of *P *< 0.05 was considered statistically significant.

## Results

### General characteristics of study population and genotype distribution of pre-screened SNPs

Table 1 presents demographic and metabolic parameters of whole study population. All the 8 SNPs which were pre-screened in 271 subjects satisfied the Hardy Weinberg Equilibrium (HWE) (P > 0.05). From the Linkage Disequilibrium (LD) test, rs174537 (*FEN1*-10154G>T) and rs174575 (*FADS*2 rs174575C>G) were found highly linked (*D' = 0.93; P < 0.001*) even though r^2 ^value was not high (r^2 ^= 0.14). rs174537 and rs2727270 (*FADS*2 rs2727270C>T) were also found highly linked (*D' = 0.99; P < 0.001*) and relatively highly correlated (r^2 ^= 0.74). Interestingly, rs174537 was found very highly linked with rs174576, rs174570 and rs174583 (*D' = 1.00, r*^*2*^*>0.95 *for all). Based on the the result above, we realized that rs174537 is sufficient to cover the genetic information of last three SNPs (rs174576, rs174570 and rs174583) as a tagging SNP. In addition, rs1000778 (*FADS*3 rs1000778C>T) is very highly linked to rs174456 (*FADS*3 rs174456T>C) (*D' = 1, r*^*2 *^*= 1*). Therefore, we finally included 4 SNPs (*FEN1*-10154 rs174537G>T, *FADS*2 rs174575C>G, *FADS*2 rs2727270C>T and *FADS*3 1000778C>T) for further genotyping in the whole study subjects.

**Table 1:** Demographic and metabolic parameters of study population

	Total (n = 567)	Range (Min-Max)
Age (year)	48.7 ± 9.3	30.0 - 69.0
Body mass index (kg/m^2^)	24.4 ± 2.7	18.2 - 35.0
Waist circumference (cm)	85.5 ± 7.3	65.0 - 127.0
Current smokers (%)	63.0	
Current drinkers (%)	16.0	
MetS (%)	17.8	
SBP (mmHg)	123.1 ± 14.3	90.0 - 180.0
DBP (mmHg)	76.9 ± 11.0	48.0 - 120.0
TG (mg/dL)	134.8 ± 73.8	27.0 - 477.0
Total-C (mg/dL)	192.2 ± 33.3	113.0 - 306.0
HDL-C (mg/dL)	50.4 ± 13.1	21.0 - 121.0
LDL-C (mg/dL)	115.1 ± 31.5	37.8 - 227.6
NON-HDL-C (mg/dL)	141.8 ± 33.9	55.0 - 266.0
ApoA1 (mg/dL)	140.5 ± 24.6	92.0 - 238.0
ApoB (mg/dL)	87.1 ± 23.5	45.0 - 162.0
Glucose (mg/dL)	92.9 ± 10.7	60.0 - 122.0
Insulin (μIU/mL)	8.9 ± 4.3	1.6 - 44.7
HOMA-IR	2.06 ± 1.1	0.4 - 11.4
Total energy expenditure (TEE, kcal)	2337.5 ± 202.5	361.0 - 2956.3
Total calorie intake (TIC, kcal)	2424.7 ± 208.2	1631.1 - 3055.6
TEE/TCI	0.965 ± 0.05	0.15 - 1.12
% of carbohydrates	61.7 ± 1.3	57.3 - 71.0
% of protein	16.9 ± 1.3	12.2 - 21.7
% of fat	21.5 ± 1.4	17.1 - 26.6
SFA(g)	8.3 ± 5.4	0.3 - 54.5
MUFA (g)	11.6 ± 7.0	0.6 - 110.3
PUFA (g)	11.3 ± 5.4	0.3 - 33.6
Cholesterol (mg)	271.2 ± 116.3	26.1 - 809.2
Fiber (g)	10.4 ± 4.7	2.5 - 39.7

Means ± S.D. or percentage

SBP: systolic blood pressure, DBP: diastolic blood pressure, TG: triglyceride, C: cholesterol, Apo: apolipoprotein, HOMA-IR (homeostatis model assessment-insulin resistance): {fasting insulin (μIU/ml) × fasting glucose (mmol/l)}/22.5, SFA: saturated fatty acid, MUFA: monounsaturated fatty acid, PUFA: polyunsaturated fatty acid

### Genotype distribution of four selected SNPs

The selected 4 SNPs which were genotyped in the whole subjects satisfied the HWE (P > 0.05) (Additional file 1). As shown in the prescreening test, rs174537 and rs174575 were found highly linked (*D' = 0.95; P < 0.001*) even though r^2 ^value was not high (r^2 ^= 0.18). rs174537 and rs2727270 were also found highly linked (*D' = 0.98; P < 0.001*) and relatively highly correlated (r^2 ^= 0.67). On the other hand, rs1000778 is weakly linked to three other SNPs (rs174537: *D' = 0.21, r*^*2 *^*= 0.04; *rs174575: *D' = 0.46, r*^*2 *^*= 0.04; *rs2727270: *D' = 0.13, r*^*2 *^*= 0.01*). The haplotype distribution of rs174537-rs174575-rs2727270-rs1000778 was like this: GCCC was most highly frequent (0.528), GCCT was second-highly frequent (0.153), TCTC was thirdly frequent (0.144) and TCTT was fourthly frequent (0.087).

Since haplotype analysis did not provide information beyond that revealed by each SNP (data not shown), we presented only the results of individual SNPs. Particularly, we presented the results especially by the form of the homozygotes for a given allele and the carrier of the alternative allele because their general and biochemical characteristics (i.e. insulin level, HOMA-IR and FA composition) were shown similarly with those among 3 genotype groups, furthermore the subject number of the minor homozygotes of rs rs174575 was so small (n = 7).

### Clinical and biochemical characteristics according to *FEN1 *-10154G>T, *FADS2 *rs174575C>G, *FADS2 *rs2727270C>T and *FADS*3 rs1000778C>T

Table 2 shows clinical and biochemical characteristics of study subjects according to the genotypes of the *FEN1 *-10154G>T, *FADS2 *rs174575C>G, *FADS2 *rs2727270C>T and *FADS*3 rs1000778C>T, respectively. No significant genotype-associated differences were observed for age, BMI, systolic blood pressure (SBP), diastolic blood pressure (DBP), serum concentrations of TG, total cholesterol (Total-C), HDL-C, LDL-C, ApoA1, ApoB (Table 2), total energy intake, proportions of energy intake derived from carbohydrates and fat in each of 4 *FADS *SNPs (Additional file 2). Interestingly, fasting insulin and HOMA-IR were significantly higher in subjects having the minor G allele of *FADS2 *rs174575C>G than those having the major CC homozygotes. On the other hand, the levels of these two markers were significantly lower in the subjects carrying the minor T allele of *FADS*2 rs2727270C>T than those having major CC homozygote (Table 3). These significant associations still maintained after adjustment for confounders (age, BMI, cigarette smoking, alcohol consumption, blood pressure, triglyceride, total energy expenditure and total calorie intake) which were followed by *Bonferroni *correction for multiple comparisons (Table 3).

**Table 2:** Demographic and metabolic parameters of study population according to *FEN1 *-10154G>T, *FADS2 *rs174575, *FADS2 *rs2727270 and *FADS*3 rs1000778C>T

	*FEN1 *-10154G>T		*FADS2 *rs174575		*FADS2 *rs2727270		*FEN1 *-10154G>T	
	
	GG (n = 259)	GT+TT (n = 308)	CC (n = 471)	CG+GG (n = 96)	CC (n = 323)	CT+TT (n = 244)	GG (n = 259)	GT+TT (n = 308)
Age (year)	48.6 ± 9.8	48.7 ± 9.0	48.9 ± 9.4	47.7 ± 9.0	48.4 ± 9.6	49.0 ± 9.0	49.1 ± 9.6	48.3 ± 9.1
BMI (kg/m^2^)	24.4 ± 2.9	24.4 ± 2.5	24.3 ± 2.7	24.8 ± 2.6	24.4 ± 2.8	24.4 ± 2.5	24.3 ± 2.6	24.5 ± 2.8
Waist (cm)	85.1 ± 6.9	85.9 ± 7.5	85.3 ± 7.3	86.8 ± 7.2	85.1 ± 7.0	86.0 ± 7.5	85.4 ± 7.3	85.6 ± 7.3
Current smokers (%)	34.7	39.0	38.0	32.3	35.3	39.3	35.7	38.3
Current drinkers (%)	83.4	84.4	83.4	86.5	84.8	82.8	79.6	88.2
Sysptolic BP (mmHg)	123.0 ± 14.7	123.2 ± 14.0	122.9 ± 14.3	124.0 ± 14.3	123.1 ± 14.7	123.2 ± 13.8	123.1 ± 13.7	123.1 ± 14.8
Diastolic BP (mmHg)	76.4 ± 11.1	77.3 ± 10.9	76.8 ± 10.9	77.4 ± 11.6	76.3 ± 11.1	77.6 ± 10.9	76.9 ± 10.5	76.9 ± 11.4
TG (mg/dL)^§^	137.9 ± 76.2	132.1 ± 71.6	133.1 ± 73.7	143.1 ± 73.8	136.7 ± 75.2	132.2 ± 71.9	135.5 ± 80.7	134.1 ± 66.5
Total-C (mg/dL)^§^	194.5 ± 32.5	190.3 ± 33.8	192.4 ± 33.3	191.4 ± 33.2	194.4 ± 33.4	189.3 ± 33.0	192.5 ± 32.3	192.0 ± 34.2
HDL-C (mg/dL)^§^	50.0 ± 12.3	50.7 ± 13.7	50.4 ± 13.3	50.2 ± 12.1	50.5 ± 12.3	50.3 ± 14.0	49.6 ± 12.9	51.1 ± 13.2
LDL-C (mg/dL)	117.2 ± 31.4	113.4 ± 31.6	115.6 ± 31.8	112.6 ± 30.3	116.8 ± 32.1	112.9 ± 30.8	116.2 ± 31.3	114.0 ± 31.8
non-HDL-C (mg/dL)	144.5 ± 33.1	139.6 ± 34.5	142.0 ± 34.2	141.2 ± 32.6	143.9 ± 33.8	139.1 ± 34.0	142.8 ± 33.7	140.8 ± 34.2
ApoA1 (mg/dL)	139.9 ± 23.0	141.0 ± 26.1	140.4 ± 24.0	140.9 ± 28.6	141.9 ± 24.5	138.9 ± 24.8	140.8 ± 26.7	140.2 ± 22.7
ApoB (mg/dL)^§^	88.6 ± 22.3	85.9 ± 24.6	87.0 ± 24.0	87.8 ± 21.3	88.6 ± 22.9	85.4 ± 24.3	85.4 ± 22.9	88.7 ± 24.1
Glucose (mg/dL)	93.3 ± 11.1	92.5 ± 10.4	92.7 ± 10.9	93.9 ± 9.8	93.4 ± 10.7	92.2 ± 10.8	92.3 ± 10.6	93.4 ± 10.9

Means ± S.D. or %, ^§^Tested by log-transformed, Performed by independent t-test or qui-square test, *P < 0.05, **P < 0.01 and ***P < 0.001 compared with major allele for each genotype.

BMI: body mass index, BP: blood pressure, TG: triglyceride, C: cholesterol, Apo: apolipoprotein, HOMA-IR(homeostatis model assessment-insulin resistance): {fasting insulin (μIU/ml) × fasting glucose (mmol/l)}/22.5.

**Table 3:** Insulin, HOMA-IR and Fatty acid composition (%) in serum phospholipids according to *FEN1 *-10154G>T, *FADS2 *rs174575, *FADS2 *rs2727270 and rs*FADS*3 1000778C>T

	*FEN1 *-10154G>T		*FADS2 *rs174575		*FADS2 *rs2727270		*FEN1 *-10154G>T	
	
	GG (n = 259)	T carrier (n = 308)	CC (n = 471)	G carrier (n = 96)	CC (n = 323)	T carrier (n = 244)	GG (n = 259)	GT+TT (n = 308)
Insulin (μIU/mL)^§^	9.1 ± 4.2	8.8 ± 4.3	8.7 ± 3.8	9.7 ± 5.9*	9.3 ± 4.8	8.4 ± 3.3*	8.6 ± 3.6	9.2 ± 4.8
HOMA-IR^§^	2.11 ± 1.04	2.03 ± 1.12	2.00 ± 0.96	2.28 ± 1.53*	2.17 ± 1.23	1.93 ± 0.83*	1.98 ± 0.91	2.15 ± 1.23
Total SFA	54.724 ± 0.340	53.694 ± 0.339*	54.374 ± 0.265	53.139 ± 0.583	54.487 ± 0.305	53.739 ± 0.389	54.039 ± 5.359	54.288 ± 6.117
12:0^§^	0.367 ± 0.014	0.411 ± 0.016	0.392 ± 0.012	0.386 ± 0.026	0.377 ± 0.013	0.410 ± 0.018	0.394 ± 0.264	0.388 ± 0.247
14:0^§^	0.595 ± 0.017	0.683 ± 0.056	0.650 ± 0.037	0.604 ± 0.031	0.599 ± 0.016	0.700 ± 0.070	0.599 ± 0.254	0.685 ± 1.019
16:0	32.736 ± 0.325	32.206 ± 0.298	32.509 ± 0.247	32.152 ± 0.474	32.666 ± 0.283	32.161 ± 0.348	32.403 ± 4.945	32.493 ± 5.514
18:0^§^	19.067 ± 0.204	18.487 ± 0.198*	18.886 ± 0.156	18.092 ± 0.348**	18.892 ± 0.186	18.566 ± 0.223	18.676 ± 3.181	18.826 ± 3.604
Total MUFA	11.353 ± 0.134	11.514 ± 0.146	11.352 ± 0.104	11.877 ± 0.297*	11.483 ± 0.134	11.385 ± 0.151	11.574 ± 2.491	11.311 ± 2.282
16:1^§^	0.628 ± 0.028	0.733 ± 0.048	0.650 ± 0.025	0.859 ± 0.117	0.656 ± 0.032	0.724 ± 0.052	0.681 ± 0.721	0.689 ± 0.655
18:1 ω-9	6.917 ± 0.101	6.996 ± 0.108	6.903 ± 0.077	7.234 ± 0.225	7.002 ± 0.103	6.903 ± 0.108	7.015 ± 1.869	6.905 ± 1.678
18:1 ω-7	1.729 ± 0.025	1.712 ± 0.027	1.715 ± 0.020	1.744 ± 0.050	1.739 ± 0.024	1.694 ± 0.029	1.755 ± 0.434	1.685 ± 0.445
Total PUFA	23.978 ± 0.377	24.046 ± 0.349	23.863 ± 0.278	24.761 ± 0.648	24.073 ± 0.343	23.939 ± 0.385	24.135 ± 5.709	23.897 ± 6.455
Total ω-6 FA	19.237 ± 0.297	19.229 ± 0.274	19.082 ± 0.217	19.973 ± 0.528	19.304 ± 0.272	19.138 ± 0.298	19.286 ± 4.365	19.180 ± 5.178
18:2 ω-6	12.062 ± 0.186	12.853 ± 0.181**	12.303 ± 0.139	13.417 ± 0.352**	12.241 ± 0.172	12.824 ± 0.200*	12.394 ± 2.794	12.587 ± 3.410
18:3 ω-6^§^	0.218 ± 0.014	0.233 ± 0.014	0.227 ± 0.012	0.225 ± 0.021	0.223 ± 0.013	0.231 ± 0.017	0.228 ± 0.258	0.225 ± 0.228
20:2 ω-6^§^	0.856 ± 0.104	0.880 ± 0.102	0.864 ± 0.082	0.898 ± 0.161	0.855 ± 0.094	0.888 ± 0.115	0.855 ± 1.790	0.883 ± 1.686
20:3 ω-6	1.504 ± 0.034	1.405 ± 0.031*	1.440 ± 0.024	1.498 ± 0.063*	1.492 ± 0.032	1.394 ± 0.033*	1.458 ± 0.513	1.442 ± 0.580
20:4 ω-6	5.016 ± 0.112	4.323 ± 0.099***	4.690 ± 0.084	4.391 ± 0.162	4.907 ± 0.100	4.285 ± 0.111***	4.806 ± 1.834	4.478 ± 1.748*
22:4 ω-6^§^	0.247 ± 0.020	0.217 ± 0.012	0.230 ± 0.012	0.232 ± 0.024	0.243 ± 0.017	0.214 ± 0.012	0.219 ± 0.191	0.242 ± 0.314
22:5 ω-6^§^	0.190 ± 0.013	0.198 ± 0.012	0.191 ± 0.010	0.209 ± 0.021	0.198 ± 0.012	0.190 ± 0.014	0.182 ± 0.194	0.206 ± 0.227
Total ω-3 FA^§^	4.741 ± 0.123	4.818 ± 0.123	4.781 ± 0.097	4.789 ± 0.199	4.769 ± 0.110	4.801 ± 0.141	4.849 ± 2.125	4.717 ± 2.031
18:3 ω-3^§^	0.135 ± 0.006	0.162 ± 0.025	0.153 ± 0.017	0.134 ± 0.009	0.134 ± 0.005	0.170 ± 0.032	0.135 ± 0.096	0.163 ± 0.460
20:5 ω-3^§^	1.158 ± 0.038	1.166 ± 0.042	1.178 ± 0.032	1.085 ± 0.062	1.161 ± 0.034	1.165 ± 0.048	1.197 ± 0.750	1.129 ± 0.602
22:5 ω-3^§^	0.515 ± 0.017	0.546 ± 0.021	0.526 ± 0.015	0.558 ± 0.036	0.523 ± 0.016	0.544 ± 0.024	0.539 ± 0.333	0.525 ± 0.312
22:6 ω-3^§^	2.833 ± 0.088	2.868 ± 0.083	2.837 ± 0.066	2.925 ± 0.146	2.852 ± 0.079	2.851 ± 0.093	2.897 ± 1.458	2.807 ± 1.404
18:3 ω-6/18:2 ω-6^§^	0.019 ± 0.001	0.020 ± 0.002	0.019 ± 0.001	0.018 ± 0.002	0.019 ± 0.001	0.020 ± 0.002	0.019 ± 0.020	0.020 ± 0.028
20:3 ω-6/18:2 ω-6	0.126 ± 0.003	0.112 ± 0.003**	0.120 ± 0.002	0.111 ± 0.004	0.123 ± 0.002	0.113 ± 0.004*	0.119 ± 0.041	0.118 ± 0.059
20:3 ω-6/18:3 ω-6^§^	13.066 ± 0.679	11.460 ± 0.541	12.206 ± 0.469	12.134 ± 1.055	12.527 ± 0.585	11.752 ± 0.626	12.487 ± 10.074	11.907 ± 10.327
20:4 ω-6/18:2 ω-6	0.419 ± 0.010	0.339 ± 0.008***	0.385 ± 0.007	0.327 ± 0.010***	0.404 ± 0.008	0.338 ± 0.010***	0.392 ± 0.157	0.359 ± 0.147**
20:4 ω-6/20:3 ω-6^§^	3.509 ± 0.102	3.192 ± 0.060**	3.383 ± 0.065	3.112 ± 0.099	3.462 ± 0.085	3.172 ± 0.069**	3.387 ± 1.020	3.288 ± 1.618*

Mean ± S.D.

^§^Tested by log-transformed, Performed by general linear model analysis (GLM, Post hoc multiple comparison tests) with adjustment for age, body mass index, cigarette smoking, alcohol drinking, blood pressure, triglyceride, total energy intake and expenditure, followed by *Bonferroni *correction for multiple comparison.

Adjusted P-value: *P < 0.05, **P < 0.01 and ***P < 0.001 compared with major allele for each genotype

### Fatty acid composition in serum phospholipids according to *FEN1 *-10154G>T, *FADS2 *rs174575C>G, *FADS2 *rs2727270C>T and *FADS*3 rs1000778C>T

Table 3 shows the FA composition in serum phospholipids according to *FEN1 *-10154G>T, *FADS2 *rs174575C>G, *FADS2 *rs2727270C>T and *FADS*3 rs1000778C>T, respectively after the adjustment for confounders (age, BMI, cigarette smoking, alcohol consumption, blood pressure, triglyceride, total energy expenditure and total calorie intake) which were followed by *Bonferroni *correction for multiple comparisons. The minor T allele carriers of *FEN1 *-10154G>T showed the higher proportion of linoleic acid (18:2ω-6, LA) (P = 0.002) and lower proportion of stearic acid (18:0, SA) (P = 0.045), dihomo-γ-linolenic acid (20:3ω-6, DGLA) (P = 0.038) and arachidonic acid (20:4ω-6, AA) (P < 0.001) in serum phospholipids than the GG homozygotes. The minor G allele carriers of *FADS2 *rs174575C>G had the higher proportion of LA (P = 0.001) and lower proportion of SA (P = 0.009) than the CC homozygotes. The minor T allele carriers of *FADS2 *rs2727270C>T showed the higher proportion of LA (P = 0.026), and lower proportions of DGLA (P = 0.035) and AA (P < 0.001) than the CC homozygtes. The minor T allele carriers of *FADS3 *rs1000778C>T had the lower proportion of AA (P = 0.022) than the CC homozygotes. Among FA ratios which indicate FA desaturase activity, AA/LA were lower in the subjects carrying the minor allele of *FEN1 *-10154G>T, *FADS2 *rs174575C>G, *FADS2 *rs2727270C>T and *FADS3 *rs1000778C>T than the major allele homozygote, respectively. DGLA/LA were also were lower in the subjects carrying the minor allele of *FEN1 *-10154G>T, *FADS2 *rs2727270C>T and *FADS3 *rs1000778C>T than the major allele homozygote, respectively. Additionally, DGLA/LA were lower in the subjects carrying the minor allele of *FEN1 *-10154G>T and *FADS2 *rs2727270C>T than the major allele homozygote, respectively.

### Correlation among fatty acid composition in serum phospholipids together with HOMA-IR

Among the FA composition in serum phospholipids, DGLA was very highly correlated with AA (r = 0.684, P =< 0.001) and LA (r = 0.533, P =< 0.001). LA was also highly correlated with AA (r = 0.550, P =< 0.001). In addition, HOMA-IR positively correlated with LA (r = 0.107, P = 0.011) and DGLA (r = 0.129, P = 0.002), and negatively with AA/DGLA (r = -0.140, P = 0.001) in total subjects. HOMA-IR also correlated with anthropometric and basic biochemical parameters such as BMI (r = 0.339, P < 0.001), waist (r = 0.305, P < 0.001), systolic blood pressure (r = 0.229, P < 0.001), diastolic blood pressure (r = 0.174, P < 0.001), triglyceride (r = 0.335, P < 0.001), total cholesterol (r = 0.083, P = 0.048), HDL-cholesterol (r = -0.197, P < 0.001), nonHDL-cholesterol (r = 0.154, P < 0.001), glucose (r = 0.4479, P < 0.001) and insulin (r = 0.967, P < 0.001).

### Association of *FADS *gene SNPs with insulin resistance according to the proportion of FA in serum phospholipids

**Figure 1:**
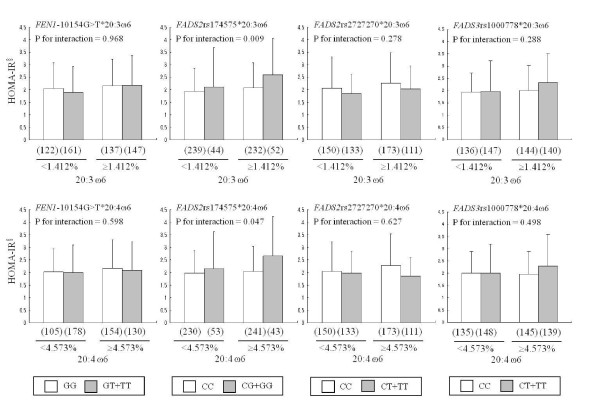
**Insulin resistance according to the proportion of dihomo-γ-linolenic acid (20:3ω-6, DGLA) or arachidonic acid (20:4ω-6, AA) in serum phospholipid and *FADS *gene polymorphisms**. Means ± S.D. (n), ^§^tested by log-transformed, *P *for interaction was tested by multiple logistic regression analysis after adjusting for age and body mass index. HOMA-IR: homeostasis model assessment of insulin resistance

Based on the correlation results between HOMA-IR and FA compositions mentioned above, study subjects were subdivided into two groups according to the proportion of LA, DGLA or AA in serum phospholipids: upper median (LA≥12.396%, DGLA≥1.412% and AA≥4.573%. of total FA composition in serum phospholipids) and lower median (LA < 12.396%, DGAL < 1.412% and AA < 4.573%). Then, we investigated the interaction effect of genetic variants and proportion of LA, DGLA or AA on their HOMA-IR levels (Figure 1). Interestingly, HOMA-IR was significantly higher in minor G allele carriers of rs174575C>G than the CC homozygotes, when the proportion of DGLA or AA were higher than median value (Interaction *P *= 0.009 and *P *= 0.047, respectively). However, significant interaction effect was neither observed with other SNPs (*FEN1 *-10154G>T, *FADS2 *rs2727270C>T or *FADS3 *rs1000778C>T) nor with LA.

### Stepwise multiple regression analysis to find the major contributors to HOMA-IR among basic parameters and *FADS *gene SNPs

Stepwise multiple linear regression analysis was performed to determine whether *FADS *gene SNPs are the independent influencing factors on HOMA-IR. Based on the results of *Pearson *correlation coefficients (between HOMA-IR and anthropometric or basic biochemical parameters) and those of the LD test, we put HOMA-IR as a dependent variable, and body mass index, triglyceride, blood pressure and 4 SNPs (*FEN1*-10154 rs174537G>T, *FADS*2 rs174575 C>G, *FADS*2 rs2727270C>T and *FADS3 *rs1000778C>T) as independent variables in the multiple stepwise linear regression model. Among the 3 SNPs, we found that *FADS*2 rs174575 C>G (β-coefficient ± S.E.: 0.253 ± 0.099, p < 0.001) remained as a significant predictor of HOMA-IR together with body mass index, triglyceride and blood pressure (R^2 ^= 0.435, P < 0.001). In addition, when we put the 4 SNPs in the model by the forms of 'the homozygotes for a given allele' (major homozygotes) and 'the carrier of the alternative allele' (heterozygotes and minor homozygotes), we found that *FADS*2 rs174575C>G (β-coefficient ± S.E.: 0.259 ± 0.110, p = 0.019) and *FADS*2 rs2727270C>T (β-coefficient ± S.E.: -0.207 ± 0.083, p = 0.013) remained as significant predictors of HOMA-IR together with other variables (R^2 ^= 0.446, P < 0.001).

## Discussion

In this present study, we found that HOMA-IR as well as serum FA composition and FA ratios were associated with the variants of *FADS *SNPs. This result is meaningful because it was first reported in Korean men but already done in Caucasians [17,18]. We also confirmed that HOMA-IR was related to FA composition in serum phospholipids, particularly LA, DGLA and AA/DGLA. Interestingly, we found the interactive effect between *FADS2 *rs174575 and the proportion DGLA or AA in serum phospholipids on HOMA-IR: *FADS2 *rs174575G allele carriers showed the remarkably higher HOMA-IR levels than the major CC homozygotes, when they had a higher proportion of DGLA (≥1.412% of total FA in serum phospholipids) or AA (≥4.573%. of total FA composition in serum phospholipids) in serum phospholipids.

So far, many studies have researched on the relationship of serum phospholipid FA composition or dietary fat with IR or metabolic disorder [1-5]. For example, Vessby and his colleague reported that people with IR or MetS had higher levels of palmitic acid and lower levels of LA in serum phospholipids [4,5]. Warensjö et al and others showed the significant positive relationship between the metabolic disorder (ie. IR and obesity) and the proportion of palmitoleic acid (16:1) and DGLA in serum phospholipids FAs [1,4,10]. They also showed the inverse association of IR with AA/DGLA which indicates the D5D activity. In our study, we found that HOMA-IR was positively associated with DGLA and negatively with AA/DGLA. However, we additionally found that HOMA-IR was positively related with LA, which is different from others. It may be related to the subject characteristics or the sources of FA. First, our study subjects were all healthy without MetS nor obesity [1,4,10]. In addition, LA and α-linolenic acid (18:3ω-3, ALA) in serum phospholipids are generally known as biomarkers of long-term essential FA intake [6-9], but palmitoleic acid and DGLA are synthesized endogenously by a sequence of desaturation and elongation reactions rather than they reflect dietary intake of those FAs [1]. It may indicate that DGLA or D5D are more relevant to explain the relationship with IR or metabolic disorder.

Recently, as examined in our study, the relationship between FA composition in serum phospholipids and the variants of *FADS *gene cluster has been studied [13-16]. Among the SNPs in *FADS *gene cluster, *FEN1*-10154G>T near *FADS*1 was reported to be most significantly associated with plasma FA composition; the minor T allele homozygotes of the *FEN1*-10154G>T are associated with lower levels of plasma AA than the major GG homozygotes [16]. In our study, the minor allele carriers of *FEN*1 -10154G>T, rs2727270C>T and *FADS3 *rs1000778C>T showed significantly lower proportion of AA. Moreover, the proportions of DGLA, DGLA/LA and AA/DGLA (D5D activity) were lower in the minor allele carriers of *FEN*1 -10154G>T and *FADS*2 rs2727270 SNPs. In addition, HOMA-IR levels were lower in the *FADS*2 rs2727270T allele carriers compared with the major CC homozytoges. All of these results were still retained after the adjustment for confounders followed by Bonferroni's correction for multiple comparisons. According to Rodriguez et al., AA or long-chain ω-6 PUFA were inversely associated with fasting insulin levels which might be due to the reduced desaturation-elongation cascade under IR condition furthermore, this phenomenon may worsen as the degree of IR increases [28]. Taken all together, our results may indicate that subjects with the major homozygotes had the impaired FA metabolism which may cause the accumulation of DGLA, and in consequence increase IR.

In addition, we found that rs2727270C>T (negative direction) and particularly *FADS2 *rs174575C>G (positive direction) were significant influent factor on HOMA-IR together with anthropometric or basic biochemical parameters. On the other hand, we found that HOMA-IR levels were higher in the *FADS*2 rs174575 G allele carriers compared with the major CC homozytoges, which were different from the results shown in *FADS*2 rs2727270C>T. These results were still retained after the adjustment for confounders followed by Bonferroni's correction for multiple comparisons. Furthermore, the correlation between HOMA-IR and serum PUFA composition were more clearly found in major C homozygotes (data not shown). Interestingly the higher HOMA-IR in *FADS2 *rs174575G carriers were more remarkable when the proportion of DGLA or AA in serum phospholipids was higher than median levels (interaction *P *= 0.009 and *P *= 0.047, respectively). These results were also still retained after the adjustment for confounders followed by Bonferroni's correction for multiple comparisons. Regarding this discordant result, we can assume that *FADS*2 rs174575 SNP differently from other *FADS *SNPs may affect the FA metabolism and that rs174575 may interact with other genes, such as the nuclear receptor peroxisome proliferator-activated receptor-γ (*PPAR-*γ) or the adipocyte C1q and collagen-domain-containing gene (*ADIPOQ*). *PPAR-*γ is a transcriptional regulator which directly interacts with the *ADIPOQ *promoter, both of them are closely associated with IR, MetS and diabetes mellitus [29-32]. It is known that PUFA acts as ligands of PPAR-γ [31], thus the pharmacologic activation with PPAR-γ agonists may lead to the increased plasma adiponectin [30,32], which may affect the glucose and insulin metabolism. This part needs to be further studied.

Our study has several limitations. First, study subjects were all healthy Korean men; thus, the results may not be applicable to women, other ethnic groups, or patients with cardiometablic syndrome whose lifestyle habits or biochemical characteristics may differ from those in our subjects. For example, Warensjö et al demonstrated that serum FA composition [i.e palmitoleic acid, γ-linolenic acid (18:3ω-6, GLA), DGLA and AA] and D6D activity were different between men and women [1]. Second, this study was performed in a cross-sectional design. It is not suitable for assessing the time sequential associations because the exposure and outcomes are collected at one point in time. Third, we need to consider the involvement of other genetic backgrounds which are associated in FA metabolism, for example *PPAR-*γ and *ADIPOQ *genes as mentioned above.

Despite these limitations, the present study shows that HOMA-IR as well as serum FA composition and FA ratios were associated with the variants of *FADS *SNPs. Furthermore, it suggested that HOMA-IR may be modulated by the interaction between the *FADS *genetic variant and the proportion of FA, particularly DGLA in serum phospholipids.

## Conclusion

HOMA-IR and serum FA composition or FA ratios were associated with the *FADS *SNPs. Interestingly, *FADS2 *rs174575 and the proportion DGLA or AA in serum phospholipids may interactively affect HOMA-IR even in healthy people.

## List of abbreviations

DGLA: dihomo-γ-linolenic acid; AA: arachidonic acid; Apo: apolipoprotein; BMI: body mass index; DBP: diastolic blood pressure; *FADS*: fatty acid desaturase; *FEN*: flapstructure specific endonuclease; HDL: high density lipoprotein cholesterol; HOMA: homeostasis model assessment; HWE: Hardy Weinberg Equilibrium; IR: insulin resistance; LA: linoleic acid; LD: Linkage Disequilibrium; LDL: low density lipoprotein; MetS: metabolic syndrome; MUFA: monounsaturated fatty acid; PUFA: polyunsaturated fatty acids; SBP: systolic blood pressure; SFA: saturated fatty acid; TCI: Total calorie intake; TEE: Total energy expenditure; TG: triglyceride

## Competing disclosure

The authors declare that they have no competing interests.

## Authors' contributions

All the authors were involved in the development of the study protocol and the experimental design. Sample collection and experiments were performed by OYK, HHL, JSC, and YJ. Data were analyzed by OYK and HHL OYK and JHL provided the research funding and wrote the manuscript. All the authors read, commented on, and contributed to the submitted manuscript.

## Supplementary Material

Additional file 1 **Genotype and haplotype distribution of study population**. It included genotype and haplotype distribution of 4 SNPs (*FEN1*-10154 rs174537G>T, *FADS*2 rs174575C>G, *FADS*2 rs2727270C>T and *FADS*3 1000778C>T) in the whole study subjects. The selected 4 SNPs satisfied the HWE (P > 0.05): minor T allele frequency of *FEN1*-10154 rs174537G>T was 0.312, minor G allele frequency of *FADS*2 rs174575C>G was 0.082, minor T allele frequency of *FADS*2 rs2727270C>T was 0.24, and minor T allele frequency of *FADS*3 1000778C>T was 0.293. This file also included the haplotype distribution of rs174537-rs174575-rs2727270-rs1000778: GCCC was the most highly frequent haplotype and the nonGCCC frequency was 0.450. Click here for file

Additional file 2 **Macro-nutrient intake and energy expenditure of study population according to *FEN1 *-10154G>T, *FADS2 *rs174575, *FADS2 *rs2727270 and rs*FADS*3 1000778C>T**. It included the information of Macro-nutrient intake and energy expenditure according to the genotypes of the *FEN1 *-10154G>T, *FADS2 *rs174575C>G, *FADS2 *rs2727270C>T and *FADS*3 rs1000778C>T, respectively. No significant genotype-associated differences were observed for total energy intake, proportions of energy intake derived from carbohydrates and fat in each of 4 *FADS *SNPs. Click here for file
