# Supplementary material for: Fatty acid desaturase (FADS) gene polymorphisms and insulin resistance in association with serum phospholipid polyunsaturated fatty acid composition in healthy Korean men: cross-sectional study
Source: Nutr Metab (Lond). 2011 Apr 23;8:24. doi: 10.1186/1743-7075-8-24 (PMC3111337; doi:10.1186/1743-7075-8-24)
Supplement: Additional file 1 — Genotype and haplotype distribution of study population. It included genotype and haplotype distribution of 4 SNPs (FEN1-10154 rs174537G>T, FADS2 rs174575C>G, FADS2 rs2727270C>T and FADS3 1000778C>T) in the whole study subjects. The selected 4 SNPs satisfied the HWE (P > 0.05): minor T allele frequency of FEN1-10154 rs174537G>T was 0.312, minor G allele frequency of FADS2 rs174575C>G was 0.082, minor T allele frequency of FADS2 rs2727270C>T was 0.24, and minor T allele frequency of FADS3 1000778C>T was 0.293. This file also included the haplotype distribution of rs174537-rs174575-rs2727270-rs1000778: GCCC was the most highly frequent haplotype and the nonGCCC frequency was 0.450. [file 1743-7075-8-24-S1.DOCX]

Additional file 1. Genotype and haplotype distribution of study population

|  | n | % |  |
| --- | --- | --- | --- |
| Genotype |  |  |  |
| FEN1 rs174537 (G>T) |  |  |  |
| G/G | 259 | 45.7 |  |
| G/T | 244 | 43.0 |  |
| T/T | 64 | 11.3 |  |
| HWE p-value | 0.785 | - |  |
| G:T frequency | 0.688:0.312 | - |  |
| FADS2 rs174575 (C>G) |  |  |  |
| C/C | 471 | 83.1 |  |
| C/G | 89 | 15.7 |  |
| G/G | 7 | 1.2 |  |
| HWE p-value | 0.735 | - |  |
| C:G frequency | 0.918:0.082 | - |  |
| FADS2 rs2727270 (C>T) |  |  |  |
| C/C | 323 | 57.0 |  |
| C/T | 211 | 37.2 |  |
| T/T | 33 | 5.8 |  |
| HWE p-value | 0.969 | - |  |
| C:T frequency | 0.76:0.24 | - |  |
| FADS3 rs1000778 (C>T) |  |  |  |
| C/C | 280 | 49.4 |  |
| C/T | 241 | 42.5 |  |
| T/T | 46 | 8.1 |  |
| HWE p-value | 0.212 | - |  |
| C:T frequency | 0.707:0.293 | - |  |
|  |  |  |  |
| Haplotype frequency |  |  |  |
| Haplotype rs174537-rs174575-rs2727270- rs1000778 | |  |  |
| GCCC/ GCCC | 159 | 28.0 |  |
| GCCC/non GCCC | 306 | 54.0 |  |
| non GCCC/non GCCC | 102 | 18.0 |  |
| GCCC: nonGCCC frequency | 0.550:0.450 | - |  |
