# Supplementary material for: Fatty acid desaturase (FADS) gene polymorphisms and insulin resistance in association with serum phospholipid polyunsaturated fatty acid composition in healthy Korean men: cross-sectional study
Source: Nutr Metab (Lond). 2011 Apr 23;8:24. doi: 10.1186/1743-7075-8-24 (PMC3111337; doi:10.1186/1743-7075-8-24)
Supplement: Additional file 2 — Macro-nutrient intake and energy expenditure of study population according to FEN1 -10154G>T, FADS2 rs174575, FADS2 rs2727270 and rsFADS3 1000778C>T. It included the information of Macro-nutrient intake and energy expenditure according to the genotypes of the FEN1 -10154G>T, FADS2 rs174575C>G, FADS2 rs2727270C>T and FADS3 rs1000778C>T, respectively. No significant genotype-associated differences were observed for total energy intake, proportions of energy intake derived from carbohydrates and fat in each of 4 FADS SNPs. [file 1743-7075-8-24-S2.DOCX]

Additional file 2. Macro-nutrient intake and energy expenditure of study population according to *FEN1* -10154G>T, *FADS2* rs174575, *FADS2* rs2727270 and rs*FADS*3 1000778C>T

|  | *FEN1* -10154G>T | | | | | | *FADS2* rs174575 | | | | | | *FADS2* rs2727270 | | | | | | *FADS3* rs1000778 | | | | | |
| --- | --- | --- | --- | --- | --- | --- | --- | --- | --- | --- | --- | --- | --- | --- | --- | --- | --- | --- | --- | --- | --- | --- | --- | --- |
|  | GG (n=259) | | | GT+TT (n=308) | | | CC (n=471) | | | CG+GG (n=96) | | | CC (n=323) | | | CT+TT (n=244) | | | CC (n=280) | | | T carrier (n=287) | | |
|  |  |  |  |  |  |  |  |  |  |  |  |  |  |  |  |  |  |  |  |  |  |  |  |  |
| TEE (kcal) | 2321 | ± | 218.7 | 2352 | ± | 187.0 | 2332 | ± | 204.3 | 2367 | ± | 191.5 | 2331 | ± | 213.1 | 2346 | ± | 187.6 | 2325 | ± | 212.4 | 2350 | ± | 191.8 |
| TCI (kcal) | 2413 | ± | 206.0 | 2435 | ± | 209.8 | 2418 | ± | 206.5 | 2458 | ± | 214.5 | 2419 | ± | 206.5 | 2432 | ± | 210.6 | 2414 | ± | 199.3 | 2435 | ± | 216.3 |
| TEE/TCI | 0.963 | ± | 0.065 | 0.967 | ± | 0.037 | 0.966 | ± | 0.054 | 0.964 | ± | 0.036 | 0.965 | ± | 0.060 | 0.966 | ± | 0.37 | 0.964 | ± | 0.060 | 0.967 | ± | 0.041 |
| % of carbohydrates | 61.6 | ± | 1.3 | 61.8 | ± | 1.4 | 61.7 | ± | 1.4 | 61.7 | ± | 1.1 | 61.7 | ± | 1.2 | 61.8 | ± | 1.5^*^ | 61.7 | ± | 1.3 | 61.7 | ± | 1.4 |
| % of protein | 17.1 | ± | 1.3 | 16.8 | ± | 1.2^*^ | 16.9 | ± | 1.3 | 16.9 | ± | 1.2 | 17.0 | ± | 1.3 | 16.8 | ± | 1.3 | 16.9 | ± | 1.2 | 16.9 | ± | 1.3 |
| % of fat | 21.5 | ± | 1.3 | 21.6 | ± | 1.4 | 21.5 | ± | 1.4 | 21.5 | ± | 1.3 | 21.5 | ± | 1.3 | 21.6 | ± | 1.4 | 21.5 | ± | 1.4 | 21.6 | ± | 1.4 |
| SFA(g) | 8.7 | ± | 5.8 | 8.0 | ± | 5.0 | 8.4 | ± | 5.6 | 8.1 | ± | 4.2 | 8.5 | ± | 5.5 | 8.0 | ± | 5.2 | 8.3 | ± | 5.3 | 8.3 | ± | 5.4 |
| MUFA (g) | 12.0 | ± | 8.4 | 11.2 | ± | 5.7 | 11.6 | ± | 7.5 | 11.3 | ± | 4.6 | 11.8 | ± | 7.8 | 11.2 | ± | 5.9 | 11.4 | ± | 6.2 | 11.6 | ± | 7.8 |
| PUFA (g) | 11.5 | ± | 5.6 | 11.1 | ± | 5.3 | 11.3 | ± | 5.5 | 11.3 | ± | 5.1 | 11.5 | ± | 5.6 | 10.9 | ± | 5.3 | 11.5 | ± | 5.5 | 11.0 | ± | 5.3 |
| Cholesterol (mg)^§^ | 270.5 | ± | 112.0 | 271.7 | ± | 120.0 | 272.4 | ± | 116.1 | 265.2 | ± | 117.8 | 267.6 | ± | 112.3 | 275.9 | ± | 121.5 | 260.6 | ± | 114.1 | 281.5 | ± | 117.7^*^ |
| Fiber (g)^§^ | 10.2 | ± | 4.6 | 10.7 | ± | 4.8 | 10.4 | ± | 4.8 | 10.6 | ± | 4.4 | 10.3 | ± | 4.6 | 10.7 | ± | 4.9 | 10.2 | ± | 4.1 | 10.7 | ± | 5.2 |

Mean±S.D. or %, ^§^Tested by log-transformed,

Performed by independent t-test or qui-square test, ^*^P<0.05, compared with major allele for each genotype.

TEE: Total energy expenditure, TCI: Total calorie intake, SFA: saturated fatty acid, MUFA: monounsaturated fatty acid, PUFA: polyunsaturated fatty acid
